# Supplementary material for: Optimizing Real-Time Vaccine Allocation in a Stochastic SIR Model
Source: PLoS One. 2016 Apr 4;11(4):e0152950. doi: 10.1371/journal.pone.0152950 (PMC4820222; doi:10.1371/journal.pone.0152950)
Supplement: S1 Text — A discussion of the algorithm used to generate the results in this paper. (PDF) [file pone.0152950.s001.pdf]

Supplementary Materials for

Optimizing Real-Time Vaccine Allocation in a Stochastic SIR Model

Chantal Nguyen & Jean M. Carlson

Algorithmic considerations

In this paper, we used a stochastic SIR framework to model the spread of an epidemic between two interacting cities, performing numerical integration with the implicit-Euler (IE) method [1]. An alternative method, Krylov subspace approximation (KSA), can also be applied to the master equation; KSA is commonly used to simplify Markov chains containing exponentials of large, sparse matrices [2]. The KSA method uses a small, dense matrix to efficiently approximate solutions to the master equation, rather than directly integrating with the generator matrix itself. The master equation can be written as the recursive series of equations:

$$\vec{P}(t_i) = \exp(\tau \mathbb{A}) \vec{P}(t_{i-1}), \tag{1}$$

which can then be solved using, for instance, the Expokit package in MATLAB [3]. The approximate solutions take the form:

$$\vec{P}(t_i) = ||\vec{P}(t_{i-1})||_2 \ \mathbb{V}(t_{i-1}) \ \exp\{\tau \mathbb{H}(t_{i-1})\} \ \hat{e}_1, \tag{2}$$

where the columns of $\mathbb{V}$ form an orthonormal basis of the m-dimensional Krylov subspace:

$$\mathcal{K}_m(t) = \text{span}\{\vec{P}(t), \tau\mathbb{A}\vec{P}(t), \ldots, (\tau\mathbb{A})^{m-1}\vec{P}(t)\}. \tag{3}$$

$\mathbb{H}$ is a dense $m \times m$ upper-Hessenberg matrix, generated using the Arnoldi method, which satisfies:

$$\mathbb{V}^{\mathrm{T}} \tau \mathbb{A} \mathbb{V} = \tau \mathbb{H}, \tag{4}$$

and $\hat{e}_1$ is the first column of the m-dimensional identity matrix. Because $m << K$, where K is the dimension of $\mathbb{A}$, the upper-Hessenberg matrix $\mathbb{H}$ is much smaller than $\mathbb{A}$.

The accuracy of the IE method is controlled by the size of the time step, which is chosen by the user; the error of the IE method is $\mathcal{O}(\tau)$. On the other hand, the accuracy of the KSA method is controlled by the size of the Krylov subspace and the Expokit error tolerance, both of which are specified by the user. Furthermore, the KSA method is prone to instabilities arising from compounded errors, and occasionally cannot produce a normalized, nonnegative probability vector without resorting to a heuristic approximation. In general, the KSA method is less computationally intensive than the IE method with the chosen time step size, but also less accurate.

A 200-day simulation of two cities with 40 people each, 0.25 coupling, and 40 vaccines administered at a 10 day delay, executed in MATLAB 8.5 on a 2.5 GHz Intel Core i7 processor, took 874 seconds with the IE method using a time step of 0.01 seconds. In comparison, the same simulation took 344 seconds with the KSA method using a Krylov subspace of dimension 65 and an Expokit error tolerance of 1×10^{-3}. An "exact" solution was generated by running the KSA method with a subspace of dimension 85 and an error tolerance of 1×10^{-7}, which took 440 seconds. The results generated with the IE and KSA methods were compared to the exact solution and had L2 errors of order 10^{-4} and 10^{-2}, respectively. While the time required to run the "exact" KSA simulation is about half that required for the IE method, the IE method allows for greater control of the integration process. The time steps are smaller and uniform in size and allow for more temporal dynamics to be extracted.

References

[1] G. Jenkinson and J. Goutsias. Numerical integration of the master equation in some models of stochastic epidemiology. *PLoS ONE*, 7, 2012.

[2] R. B. Sidje and W. J. Stewart. A numerical study of large sparse matrix exponentials arising in markov chains. *Comput Stat Data An*, 29, 1999.

[3] R. B. Sidje. Expokit: A software package for computing matrix exponentials. *ACM T Math Software*, 24, 1998.
