# Supplementary figures and images for: Optimizing Real-Time Vaccine Allocation in a Stochastic SIR Model
Source: PLoS One. 2016 Apr 4;11(4):e0152950. doi: 10.1371/journal.pone.0152950 (PMC4820222; doi:10.1371/journal.pone.0152950)

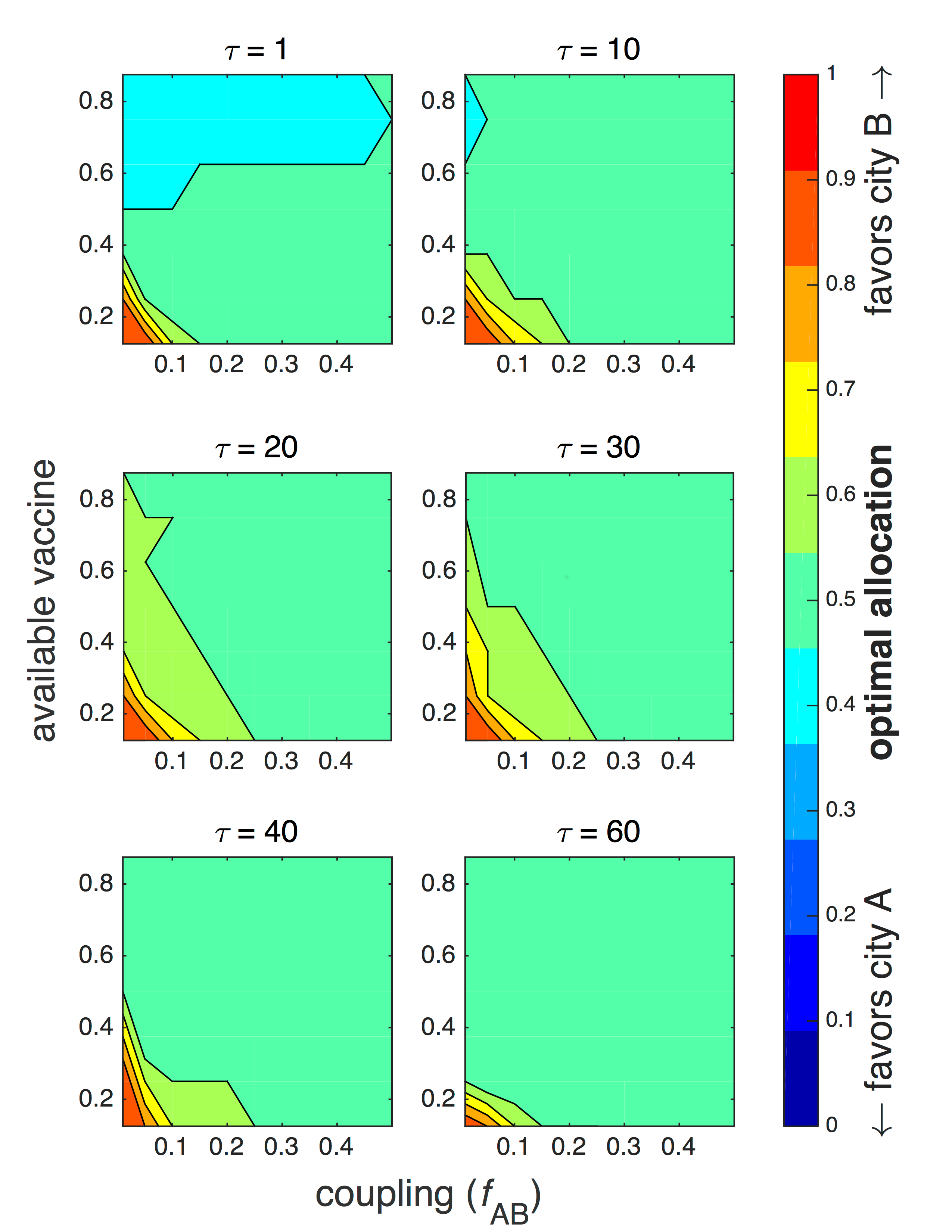

Supplement: S1 Fig — The optimal fraction of total vaccine allocated to city B in the deterministic model is plotted as a function of available vaccine (expressed as a fraction of the total combined population) and coupling fAB for different fixed values of time delay τranging from 1 to 60 days. (TIFF) [file pone.0152950.s003.tiff]

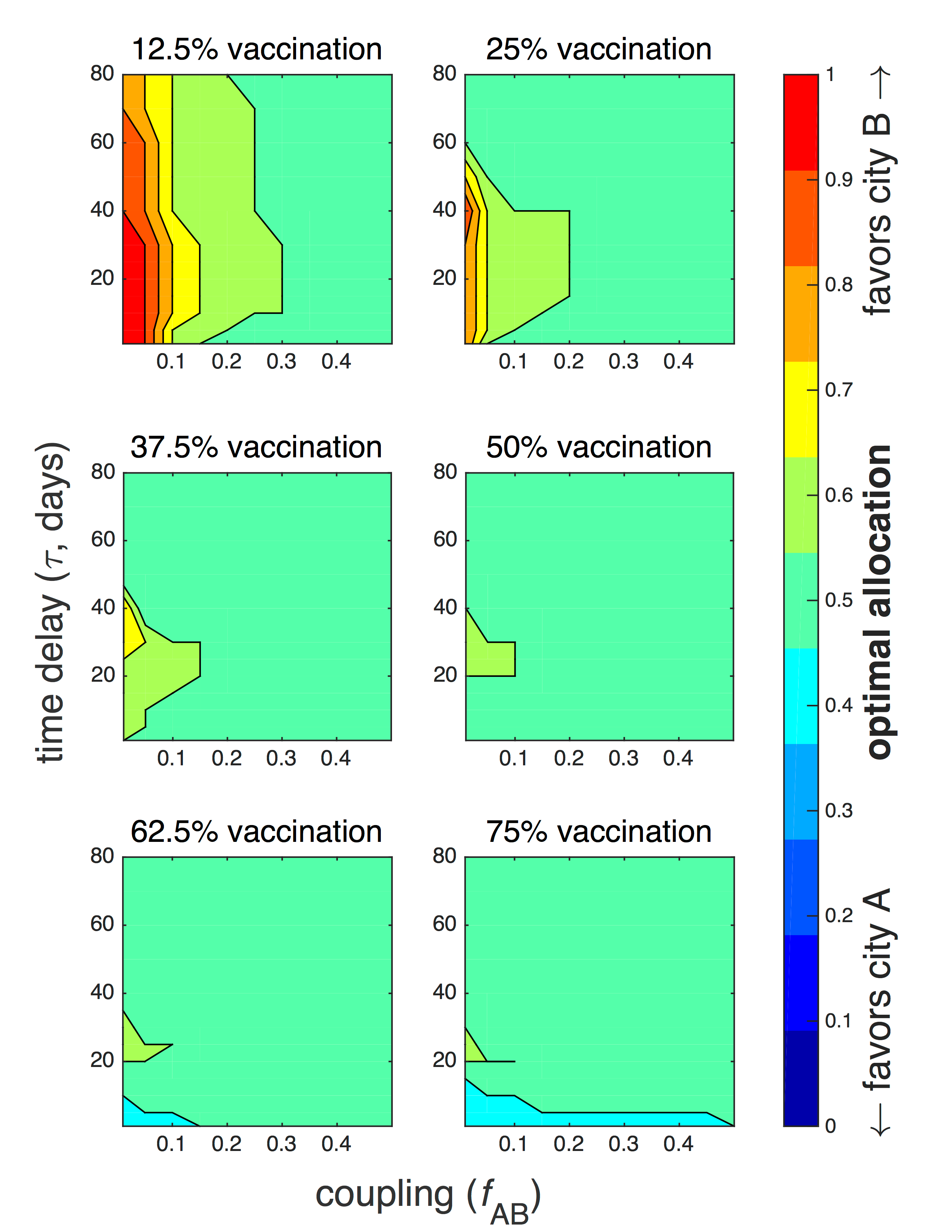

Supplement: S2 Fig — Optimal fraction of total vaccine allocated to city B in the deterministic model plotted as a function of time delay τ and coupling fAB for different fixed values of available resources ranging from 10 to 60 vaccines (i.e. 12% to 75% vaccination). (TIFF) [file pone.0152950.s004.tiff]

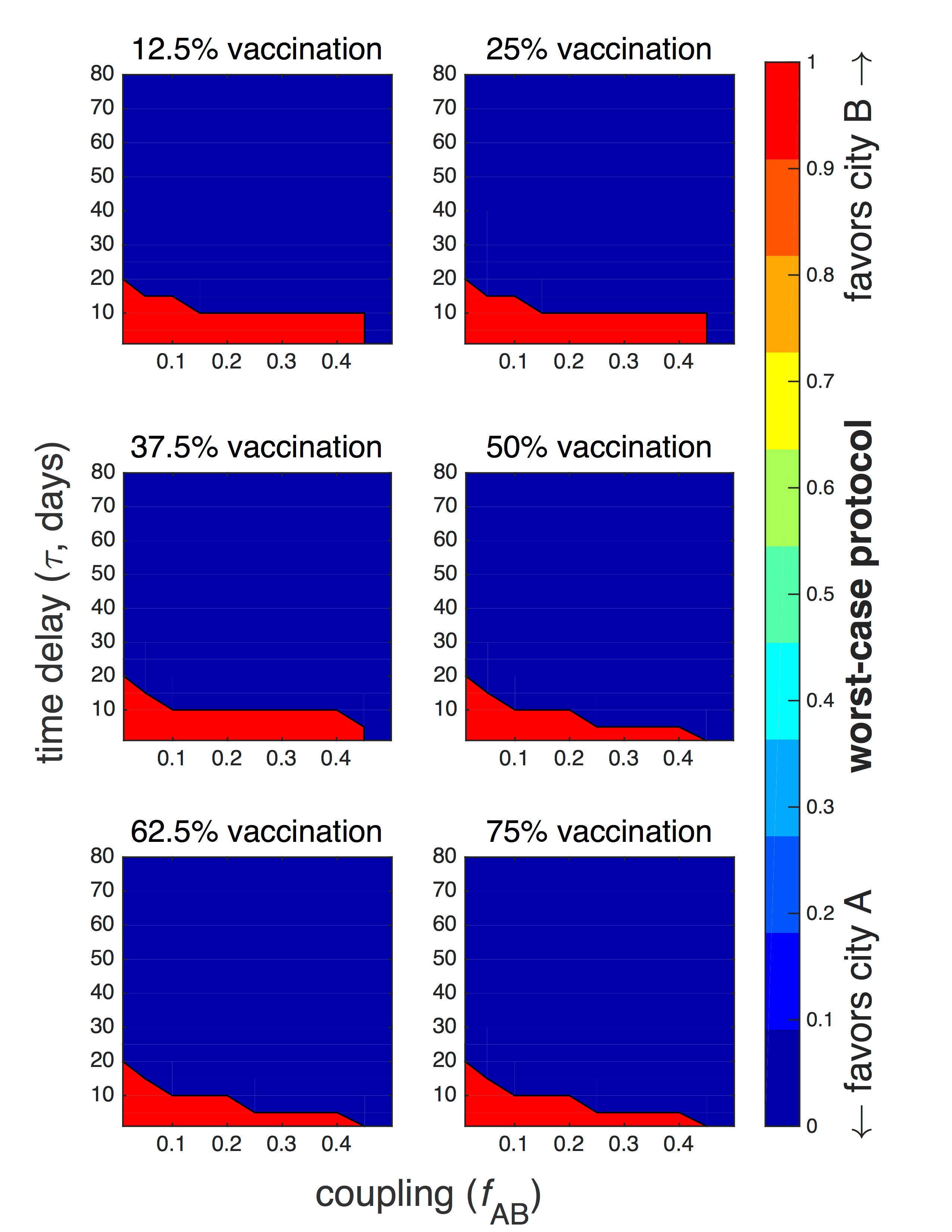

Supplement: S3 Fig — The vaccine allocations resulting in maximum mean final epidemic size 〈E〉max are plotted as a function of time delay τ and coupling fAB for different fixed amounts of available vaccine. City A has 39 initial susceptibles and one infective; city B has 40 initial susceptibles. The recovery rate γ = 0.15 and the reproductive number r0 = 2. (TIFF) [file pone.0152950.s005.tiff]

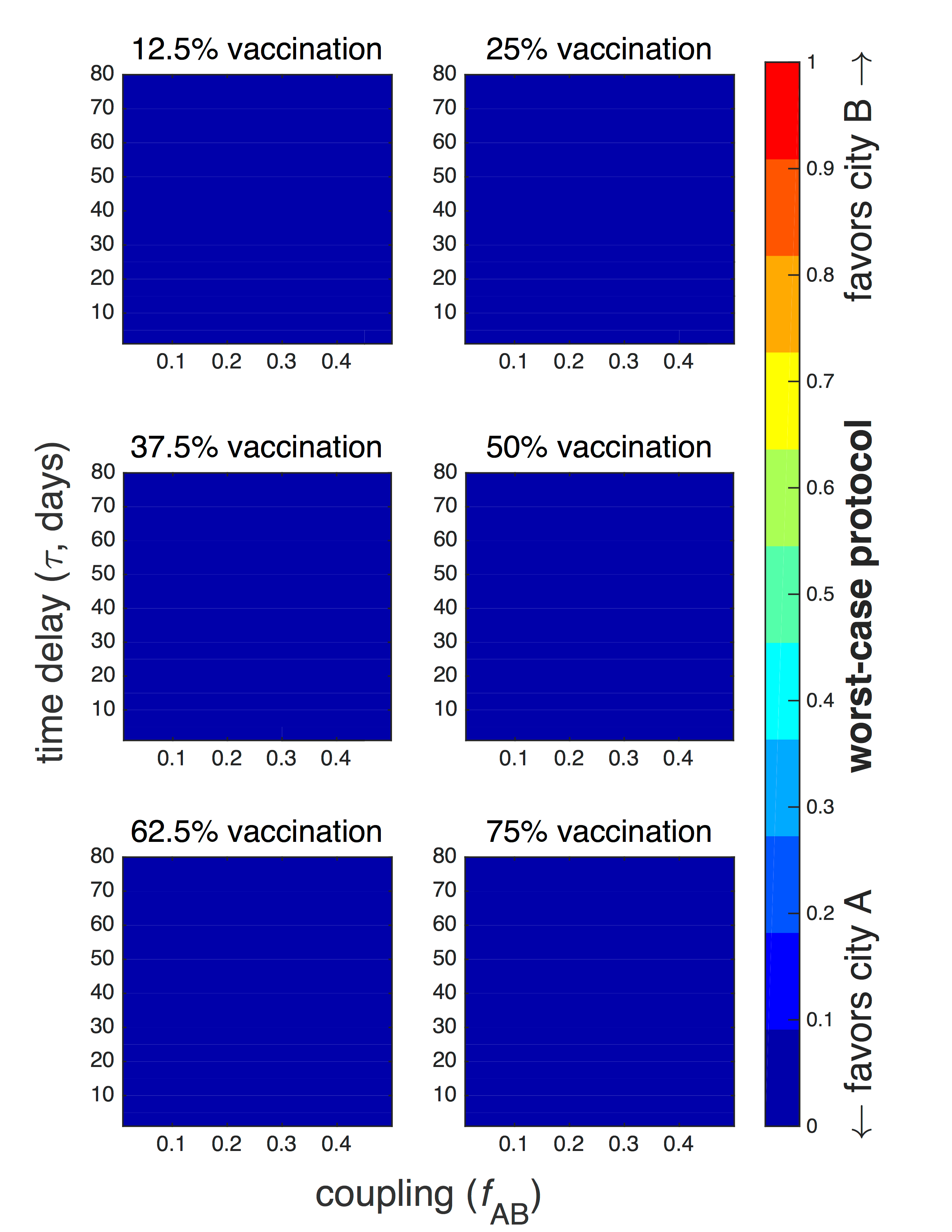

Supplement: S4 Fig — The vaccine allocations resulting in maximum final epidemic size Edetmax are plotted as a function of time delay τ and coupling fAB for different fixed amounts of available vaccine. City A has 39 initial susceptibles and one infective; city B has 40 initial susceptibles. The recovery rate γ = 0.15 and the reproductive number r0 = 2. (TIFF) [file pone.0152950.s006.tiff]

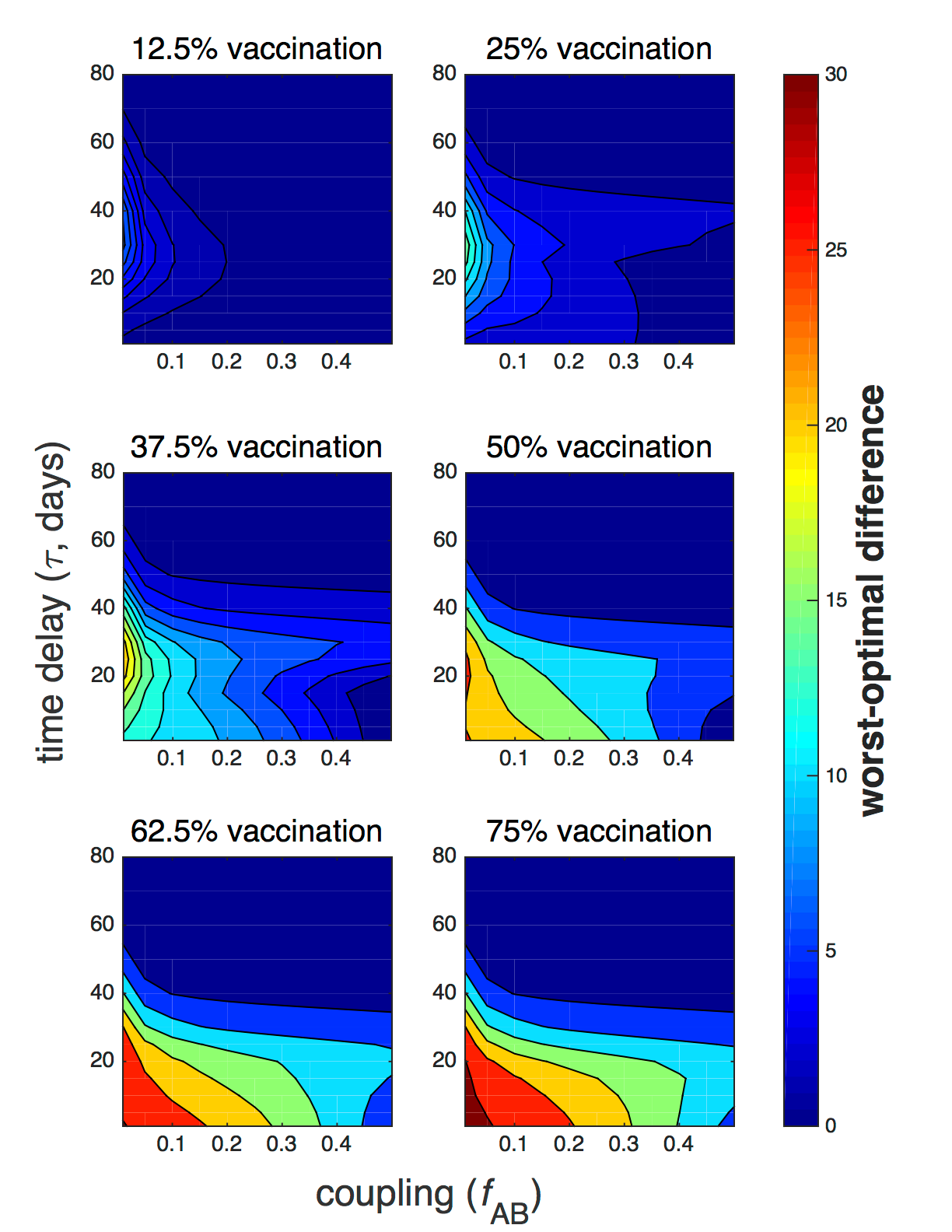

Supplement: S5 Fig — The difference in deterministic final epidemic size Edet between worst-case and optimal protocols is plotted as a function of time delay τ and coupling fAB for different fixed amounts of available vaccine. (TIFF) [file pone.0152950.s007.tiff]
